# De-escalation of critical care and prevention of iatrogenicity through a self-tracking daily rounding checklist

**DOI:** 10.3389/fped.2026.1746558

**Published:** 2026-03-10

**Authors:** Oluwatomini A. Fashina, Robert J. Kahoud, Sheri S. Crow, Grace M. Arteaga, Yu Kawai

**Affiliations:** 1 Department of Pediatrics, Mayo Clinic Children’s, Rochester, MN, United States; 2 Department of Pediatrics, Division of Pediatric Critical Care Medicine, Mayo Clinic Children’s, Rochester, MN, United States

**Keywords:** ABCDEF bundle, critical care de-escalation, iatrogenicity, patient safety, pediatric critical care, quality, rounding checklist

## Abstract

**Introduction:**

As part of the Society of Critical Care Medicine's Pediatric ICU Liberation Campaign Collaborative, Mayo Clinic developed a daily physician-led rounding checklist to promote timely de-escalation of ICU support and proactive measures to prevent iatrogenic conditions. We hypothesized that implementing this checklist would be feasible and would reduce missed opportunities to optimize care through both de-escalation and initiation of key preventive tasks.

**Methods:**

We conducted a prospective study in a 16-bed medical/surgical PICU at a quaternary academic center over a 300-day period from May 2017 to March 2018. A 15-task daily rounding checklist was integrated into standard patient-centered rounds for every admitted patient to prompt physicians to address common de-escalation and preventive care tasks. For each task, physicians documented whether it had already been addressed during rounds, was not applicable to the patient, or the checklist directly prompted a plan modification. Trends in checklist compliance and the frequency/type of plan modifications were assessed in 60-day intervals to evaluate sustainability over time.

**Results:**

1,710 rounding checklists were completed across 2,424 patient-days (71% compliance). The checklist prompted 198 documented plan modifications from 9% of all completed checklists. The most frequent modifications involved initiating a bowel regimen (*n* = 47), converting intravenous to enteral medications (*n* = 32), initiating gastrointestinal prophylaxis (*n* = 30), consulting rehabilitation services (*n* = 18), and restarting home medications (*n* = 18), which together accounted for 73% (145/198) of all modifications.

**Discussion:**

Implementation of a daily rounding checklist was feasible, achieved acceptable compliance, and identified 198 opportunities for timely de-escalation and proactive care in a complex PICU environment. Although overall compliance did not meet the *a priori* >75% target across the entire study period, early adoption exceeded this threshold and later decline highlighted sustainability challenges. This initiative demonstrates the potential of structured physician-led checklists to standardize care and reduce iatrogenic risk in pediatric critical care.

## Introduction

Intensive care unit (ICU) iatrogenicity remains both persistent and pervasive (1). Hospital-acquired conditions, such as gastrointestinal (GI) bleed, venous thromboembolism (VTE), delirium, immobility, and related complications, are prevalent in adult and pediatric ICUs alike (2). Decisions surrounding sedation, mechanical ventilation, pain control, mobilization, and prophylactic measures can all contribute to these unintended harms (1, 3, 4). To mitigate this, there is a growing call for standardized protocols that emphasize clear communication and interdisciplinary care, both to de-escalate unnecessary ICU interventions and to initiate key preventive practices, all with the shared goal of minimizing iatrogenicity.

Recent publications from the SCCM's PICU Liberation Quality Improvement Collaborative highlight the value of interdisciplinary interventions in improving healthcare outcomes, emphasizing the need for further evaluation of tools that integrate both de-escalation and proactive care (3). The medical team in our PICU felt that a rounding checklist to support timely weaning of ICU support and the proactive introduction of measures, such as a GI and VTE prophylaxis, bowel regimens, sleep enhancement protocols, and early rehabilitation consults [physical therapy (PT), occupational therapy (OT) and/or physical medicine & rehabilitation (PM&R)], would improve care.

The goal of this study is to determine the feasibility and impact of developing and implementing a physician-led rounding checklist to support the de-escalation of critical care and the initiation of proactive measures aimed at preventing iatrogenicity. Mirroring the principles of SCCM's ABCDEF Pediatric Intensive Care Unit (PICU) Liberation bundle, it was designed to be adaptable and applicable for daily use in all critically ill infants and children, regardless of the level of medical complexity and/or mechanical support (3). We hypothesized that compliance with the checklist use would be acceptable (>75%) and that the frequency of medical plan modification would be higher during the first months compared to the last months of the study, given unit culture improvement from the introduction of consistent reminders to perform tasks**.**

## Methods

### Ethics

We conducted a single-center prospective study in the PICU at Mayo Clinic Children's. The study was approved by the Mayo Foundation for Medical Education and Research Institutional Review Board (IRB No. 16-004000) on July 13, 2016. Individual informed consent was waived. All aspects of the study adhered to the ethical principles outlined in the Declaration of Helsinki (1975, revised in 2000).

### Study design

**Figure 1:**
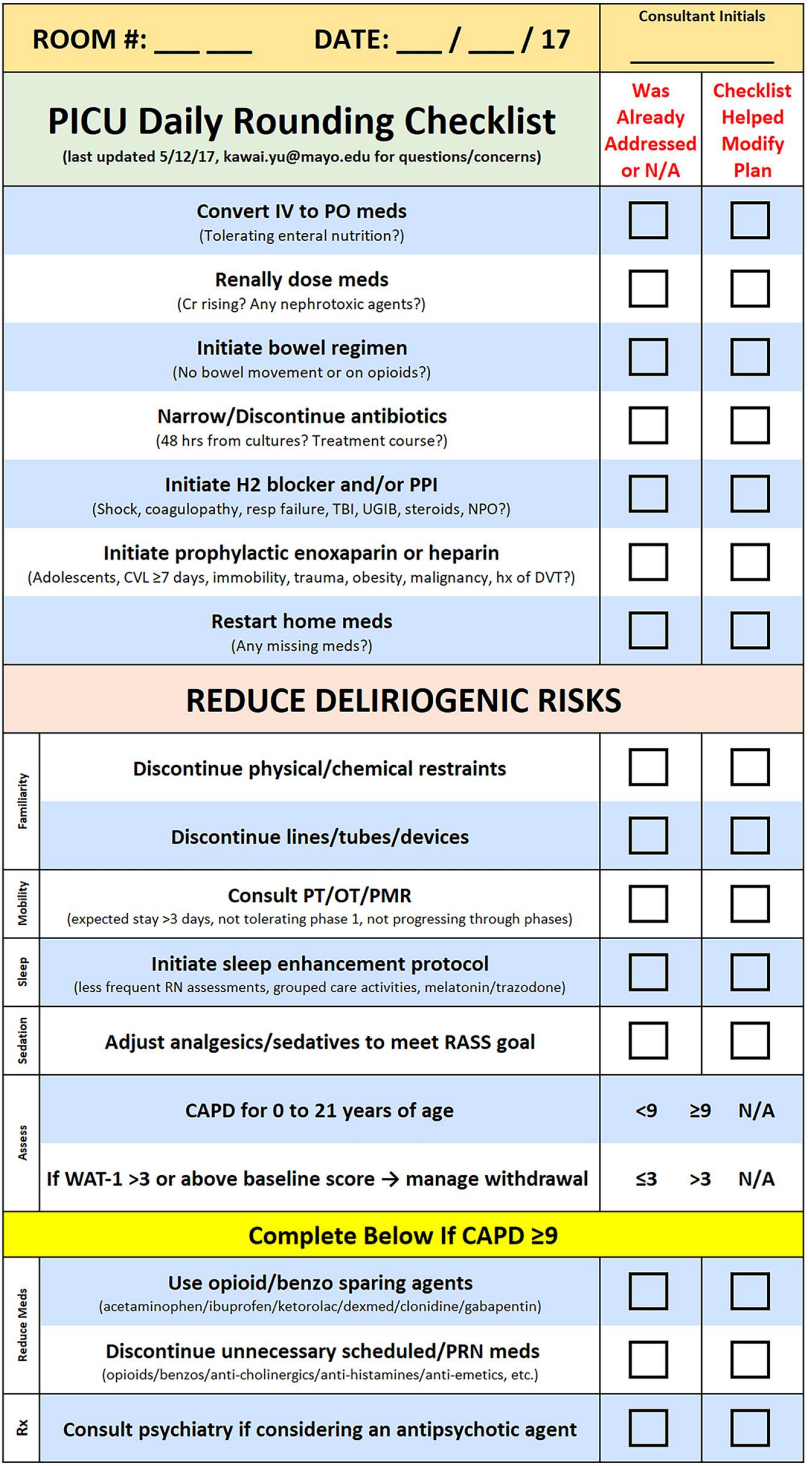
Daily rounding checklist.

This initiative was conducted over 300 days (May 2017—March 2018) in a 16-bed medical/surgical PICU at a quaternary academic center. Prior to implementation, daily medical rounds did not utilize a checklist. To address persistent iatrogenic risks, a structured, physician-led rounding checklist was created to guide both the de-escalation of critical care interventions and the initiation of proactive measures (Figure 1). The checklist was developed through a comprehensive literature review and multidisciplinary input (physicians, bedside nurses, pharmacists, dietitians, physical therapists, and occupational therapists). Educational materials and written guidance were distributed before implementation to ensure familiarity. During rounds, the paper checklist was integrated toward the end of the medical team's presentation for each patient.

The structured daily rounding checklist included 15 tasks:

Converting intravenous (IV) to enteral medications
Adjusting medication dose based on renal clearance
Initiating a bowel regimen
Narrowing or discontinuing systemic antibiotics
Initiating GI prophylaxis
Initiating VTE prophylaxis
Restarting home medications
Discontinuing physical and chemical restraints
Discontinuing lines, tubes, and/or devices
Consulting PT, OT, and/or physical medicine and rehabilitation consultative service
Initiating a sleep enhancement protocol
Adjusting analgesic/sedative medications to meet Richmond Agitation Sedation Scale (RASS) goal (5)
Utilizing opioid and benzodiazepine-sparing analgesic and sedative medications
Discontinuing unnecessary scheduled or as needed medications
Consulting a pediatric psychiatry team if considering an antipsychotic medication to treat delirium

Tasks 13–15 were only reviewed if the Cornell Assessment of Pediatric Delirium (CAPD) score was 9 or higher, suggesting that the patient may have delirium (5). CAPD screening was part of routine clinical practice in our unit; the local estimate that approximately 30%–40% of patients/patient-days have at least one positive CAPD screen reflects our unit's typical delirium screening experience and is consistent with published pooled prevalence estimates (6).

The flow of medical rounds was standardized, and a summary was sent to medical residents, bedside nurses, physicians, dietitians, pharmacists, and respiratory therapists (Supplementary Figure S1). In brief, the primary medical resident will begin presenting the patient on rounds, and during the presentation, a second resident will complete the checklist. If the checklist task was discussed during the presentation, the secondary resident will check off the “Already Addressed” box. At the end of the resident presentation, if any tasks remain that were not discussed, the secondary resident will verbalize them. If the verbalized task does not apply to the patient, then the “Not Applicable” box was checked off. If the patient's plan was modified by verbalizing the task, then the “Checklist Helped Modify Plan” box was checked.

### Data collection

The rounding checklist self-tracked its impact during medical rounds by allowing physicians to indicate for each task whether it was: “Already Addressed” during resident presentation of a patient, “Not Applicable” to the patient, or if the “Checklist Helped Modify Plan” for the patient, as previously described. Completed checklists were collected weekly throughout the 300-day period. The daily PICU census, used to determine eligible patient days, was obtained at 7 a.m. every morning. Medical rounds typically start at 8:30 a.m. and can last until 10:30–11 a.m.

### Outcome measures

The main outcome was overall physician compliance with daily checklist use. Compliance was calculated as the number of completed checklists collected divided by the eligible patient-days. The other outcome was the number and distribution of plan modifications prompted by the checklist, reflecting opportunities to de-escalate care or add preventive interventions.

### Statistical analysis

Data were summarized descriptively. Compliance rates and modification frequencies were calculated for the entire 300-day period and examined in 60-day intervals (1–60, 61–120, 121–180, 181–240, 241–300 days) to evaluate trends and sustainability over time. Plan modifications were analyzed to determine which tasks most frequently influenced clinical plans. Analyses were intended to describe observed patterns rather than to prove causation.

## Results

Over the 300-day implementation period, the 15-task daily rounding checklist was completed on 1,710 out of 2,424 eligible patient-days, yielding an overall compliance rate of 71% (Table 1). Compliance was highest in the early months: 78% during days 1–60, peaking at 88% during days 61–120, and then tapering to 67% in days 121–180, 67% in days 181–240, and 53% in days 241–300. The checklist prompted a total of 198 documented medical plan modifications over the 300 days, resulting in 9% of completed checklists leading to changes in the patients' daily plans (Table 1). 14% of completed checklists led to a plan modification in days 1–60, 5% in days 61–120, 11% in days 121–180, 5% in days 181–240, and 6% in days 241–300.

**Table 1:** Self-tracking rounding checklist compliance and task modification over 300 days.

Measure	1–60 days	61–120 days	121–180 days	181–240 days	241–300 days	1–300 days
PICU Census (*n*)	467	489	449	517	502	2,424
Completed Checklists (*n*)	363	432	302	346	267	1,710
Compliance (%)	78%	88%	67%	67%	53%	71%
Total Modified Tasks (*n*)	67	26	41	20	44	198
Top 5 Most Frequently Modified Tasks and their Proportion (%) within the Total Modified Tasks	Bowel regimen	GI prophylaxis	Bowel regimen	Bowel regimen	IV to PO conversion	Bowel regimen
	2. IV to PO conversion	2. Bowel regimen	2. GI prophylaxis	2. Home medications	2. Sleep enhancement	2. IV to PO conversion
	3. GI prophylaxis	3. Antibiotics	3. IV to PO conversion	3. PT/OT	3. VTE prophylaxis	3. GI prophylaxis
	4. PT/OT	4. Psychiatry	4. Home medications	4. GI prophylaxis	4. GI prophylaxis	4. Home medications
	5. Sleep enhancement	5. IV to PO conversion	5. Lines/tubes/ devices	5. IV to PO conversion	5. PT/OT	5. PT/OT
	79%	77%	80%	95%	66%	73%
Checklist Proportion (%) Leading to Any Modifications	14%	5%	11%	5%	6%	9%

PICU = Pediatric Intensive Care Unit; n = absolute count; % = percentage; Bowel regimen = initiate a bowel regimen; IV to PO conversion = convert intravenous medications to oral medications; GI prophylaxis = initiate medications for gastrointestinal prophylaxis; PT/OT = consult rehabilitation services; Sleep enhancement = initiate sleep enhancement measures; Antibiotics = narrow or discontinue antibiotics; Psychiatry = consult psychiatry if considering antipsychotics; Home medications = reinitiate home medications; Lines/tubes/devices = discontinue lines, tubes and/or devices; VTE prophylaxis = initiation medications for venous thromboembolism prophylaxis.

The 5 most frequently prompted checklist tasks accounted for 73% (145/198) of all plan modifications over the 300 days, with this distribution remaining similar across each 60-day time periods: 79% of modifications in days 1–60, 77% in days 61–120, 80% in days 121–180, 95% in days 181–240, and 66% in days 241–300, respectively. The five most frequently modified tasks were: initiating a bowel regimen (*n* = 47), converting IV to enteral medications (*n* = 32), initiating GI prophylaxis (*n* = 30), consulting physical therapy/occupational therapy/physical medicine and rehabilitation services (*n* = 18), and restarting home medications (*n* = 18).

Other tasks—such as initiating a sleep enhancement protocol (*n* = 15), initiating VTE prophylaxis (*n* = 14), discontinuing unnecessary lines, tubes, or devices (*n* = 8), narrowing or discontinuing antibiotic therapy (*n* = 5), and adjusting medication dose based on renal clearance (*n* = 5) were less frequently modified. The tasks that were only reviewed when CAPD was positive, including utilizing opioid and benzodiazepine-sparing analgesic and sedative medications (*n* = 1), discontinuing unnecessary scheduled or as-needed medications (*n* = 1), and consulting pediatric psychiatry if considering an antipsychotic medication to treat delirium (*n* = 4), had even less impact on patient plan modifications. The tasks that did not lead to any plan modification were discontinuing physical and chemical restraints and adjusting analgesic/sedative medications to meet the RASS goal.

## Discussion

This study presents the first single-center experience of a self-tracking, physician-led, medical rounding checklist designed to reduce iatrogenic harm and simultaneously de-escalate care in a Pediatric Intensive Care Unit. The unique, self-tracking element enabled the team to capture, in real-time, which tasks directly modified care plans. Designed to promote the timely weaning of ICU interventions and activate preventive strategies, the paper checklist proved feasible for daily use, achieving 71% overall compliance over a 300-day period, with compliance exceeding the predefined 75% feasibility threshold during early implementation but declining over time, and resulting in 198 medical plan modifications. This supports its feasibility and measurable impact on daily clinical care.

Most frequently, checklist-driven plan modifications involved initiating bowel regimens, starting GI prophylaxis, converting IV to enteral medications, restarting home medications, and initiating mobilization through rehabilitation consults. These reflect common but easily overlooked opportunities where ICU care can be streamlined and ICU liberation optimized.

Importantly, many checklist domains are interdependent. Sedation practices influence delirium risk, sleep quality, and mobilization, while early rehabilitation and sleep promotion may mitigate cognitive and functional decline. By prompting systematic review of these domains during rounds, the checklist reinforced their interconnected role in minimizing iatrogenic harm and optimizing recovery.

In contrast, specific tasks produced few or no modifications for identifiable reasons. The final three checklist tasks, utilizing opioid- and benzodiazepine-sparing agents, discontinuing unnecessary medications, and consulting psychiatry for antipsychotic consideration, collectively led to only six plan modifications over 300 days. This likely reflects that these tasks were conditionally reviewed only when the CAPD score was positive. Because the CAPD-positive rate in our unit averages 30%–40%, this conditional structure may have limited the frequency with which these tasks were reviewed. They were included in this way to maintain checklist brevity while ensuring delirium-specific measures were addressed for high-risk patients. Similarly, the task, “discontinuing chemical or physical restraints”, produced no plan modifications, as restraint use in our PICU is low and already subject to daily medical necessity review per unit policy, rendering the checklist prompt largely redundant. The task, “adjust analgesic/sedative medications to meet RASS goal”, also did not result in modifications, likely because it is routinely addressed within the nursing rounding script, where nurses report each patient's current RASS goal, recent RASS score, and ongoing analgesic and sedative infusions.

Notably, the inclusion of these consistently addressed tasks underscores another value of a self-tracking checklist: identifying which components of ICU care are already reliably integrated into team workflow vs. those that benefit from explicit prompting. The checklist tasks can be reviewed regularly to create a more efficient tool that fits the unit's needs. We did not modify the checklist during the 300-day period to obtain consistent data, but we recognize this benefit of utilizing the self-tracking checklist.

Beyond patient-level care optimization, such checklists can also serve as valuable performance improvement tools. Because each task is documented in real-time, institutions can adapt this framework to track provider or team adherence to evidence-based practices for Joint Commission or departmental quality metrics. Given the shift-based nature of critical care and the shared responsibility among multiple attending physicians, having a structured, auditable process offers an equitable and reproducible method for evaluating consistency of care delivery.

While compliance and modification rates gradually declined over time, this pattern likely reflects multiple factors, including checklist fatigue, workflow pressures, staff and trainee turnover, and variability in attending adoption. The persistence of checklist use and the continued frequency of high-impact modifications suggest partial assimilation of ICU liberation principles into routine practice rather than loss of clinical relevance. This decline likely reflects increased awareness among healthcare professionals and the habitual inclusion of checklist tasks into routine care, leading to less dependence on the checklist rather than a reduced utility of the tool itself. The fact that even on days 241–300, when compliance with the checklist utilization was its lowest at 53%, 44 total tasks were modified, highlights the ongoing need for a checklist to remind the medical team regarding commonly forgotten tasks. However, sustaining the incorporation of a new task into the daily workflow can be challenging.

Iatrogenic harm in the PICU remains a significant contributor to morbidity and prolonged critical illness (1). Hospital-acquired conditions, particularly immobility, over-sedation, delirium, and withdrawal syndromes, continue to impact critically ill infants and children (1). Decisions regarding sedation, mechanical ventilation, pain management, and mobilization can unintentionally perpetuate this cycle (1, 3, 4). More pediatric-centered research is needed to define and address ICU-related iatrogenicity and optimize recovery trajectories and/or prevention strategies.

The SCCM's Pediatric ICU Liberation Campaign Collaborative is a quality improvement initiative that focuses on the comprehensive interprofessional care necessary to ensure the integration of liberation and de-escalation in the care of critically ill infants and children (3). It represents a growing effort to address this issue through structured, multidisciplinary interventions. Initially designed for adult ICUs, the ABCDEF bundle and related practices have since been adapted for pediatric settings (3). The first multicenter study offering a comprehensive evaluation of ABCDEF bundle utilization in pediatric critical care was published in 2023 (3). Yet, despite this momentum, the pediatric literature remains sparse, particularly regarding standardized de-escalation strategies.

This study offers a pragmatic contribution to the field by translating ICU liberation principles into a physician-led, task-based checklist that can be used across all pediatric patients, regardless of complexity or support level. Unlike interventions focused solely on sedation or mobility, this checklist provides a comprehensive review of iatrogenic risks, including medication overuse, under-mobilization, line/device dependency, and lack of prophylaxis or supportive care.

Several prior studies have examined the impact of iatrogenic conditions in pediatric critical care, emphasizing their prevalence and preventability. Research on opioid and benzodiazepine withdrawal has highlighted the frequency of iatrogenic withdrawal syndromes and their association with prolonged sedation and hospitalization (7, 8). Other investigations have shown that iatrogenic events, including infections and management errors, remain a significant cause of PICU admissions and morbidity (9, 10). Collectively, these studies emphasize the need for strategies that proactively prevent iatrogenicity during ongoing critical care rather than reacting to isolated complications after they occur.

Building on this, recent efforts have evaluated structured checklists as tools to improve consistency, safety, and communication in the PICU environment. Cifra et al. introduced a best-practice rounding checklist to enhance adherence to daily care goals, while Gardner et al. standardized multidisciplinary rounds to strengthen communication and reduce safety events (11, 12). Jones et al. implemented the “Glass Door” goals checklist to improve team coordination and family engagement (13). Van Dijk et al. proposed the *mosaIC* checklist to integrate pain, withdrawal, and delirium assessments into a single framework, and Geva et al. developed *eSIMPLER*, an electronic, EHR-integrated checklist for real-time decision support during rounds (14, 15).

Together, these studies underscore the value of checklists in improving process reliability and interdisciplinary communication but highlight an ongoing gap in the literature: few have targeted a systems-level framework that unites both de-escalation and prevention across all critically ill children. The present initiative addresses this gap by providing a self-tracking, physician-led checklist designed to integrate ICU liberation principles directly into daily workflow and clinical decision-making.

### Limitations

Despite these promising results, several limitations should be acknowledged. First, this was a single-center study without a formal pre-intervention comparison group, limiting causal inference. While the frequency of plan modifications provides an indirect measure of clinical impact, the study did not assess patient-centered outcomes. We did not assess clinical endpoints such as ICU length of stay, incidence of delirium or withdrawal, and rate of hospital-acquired complications (e.g., GI bleed, constipation, critical illness myopathy, central-line associated bloodstream infection, catheter-related urinary tract infection, or venous thromboembolism). These outcomes would be important in future studies; however, given the time elapsed since data collection and the absence of standardized, reliably extractable baseline outcome data across the full cohort, we were unable to retrospectively construct a valid pre-post outcome comparison.

The study did not record the exact time required to complete the checklist; however, because the non-presenting resident completed it concurrently during rounds and only verbalized tasks not already addressed, completion likely added less than one minute per patient. Among the six attending physicians who utilized the checklist, adoption varied. For example, two rotated infrequently through the PICU and were slower adopters. When excluding these rotations, overall compliance approached 80%, suggesting that institutions with core attending coverage may achieve even higher adherence. Resident use of the checklist may also have introduced variability and cognitive burden, particularly among junior trainees who may have limited experience determining the clinical relevance of specific checklist items. In addition, assigning residents to complete the checklist may risk shifting their role toward documentation and away from active clinical learning. Alternatively, by reviewing the checklist content, residents gain increased exposure to ICU liberation and important de-escalation tasks. Misclassification of tasks could have led to over- or underestimation of plan modification frequency. Alternative workflows, including nurse-driven, pharmacist-driven, or multidisciplinary EHR-integrated checklists, may reduce trainee burden and improve reliability across diverse unit structures. Moreover, because the checklist was paper-based and relied on manual collection, occasional loss of forms likely contributed to an underestimation of compliance.

Additionally, the PICU census was recorded at 7:00 a.m., while rounds typically occurred between 8:30 a.m. and 10:30 a.m.; if patients were temporarily unavailable (e.g., off the unit for procedures) or permanently left the unit (transferred to a different unit or discharged home) before 10:30 a.m., checklist completion could not occur, further underestimating true compliance.

Furthermore, although checklist use was sustained across the 300-day period, adherence decreased in later months. This may reflect checklist fatigue, staffing turnover, or diffusion of checklist elements into unstructured workflows, possibilities that merit further qualitative exploration. Plan-Do-Study-Act (PDSA) cycles were not performed during this initial implementation period; however, they will form the basis of future work grounded in implementation-science principles. Incorporating real-time digital tools or automated reminders within the electronic health record may also enhance sustainability.

While elements of observational research were incorporated for structure and transparency, this work was primarily a quality improvement initiative rather than traditional scientific research. The primary objective was to assess the feasibility of developing a consistent and reproducible workflow that integrates key principles of safe pediatric critical care. Because the implementation team also designed and used the checklist, some degree of inherent bias is possible; however, the intent was not to test a hypothesis but to enhance and standardize care processes within a single-center ICU setting.

Overall, these results support the feasibility and potential utility of a daily physician-led rounding checklist in a quaternary academic PICU. By capturing both de-escalation opportunities and proactive measures, the checklist served as a practical, low-burden tool to standardize care processes and potentially reduce iatrogenic harm in a complex PICU environment. Future multicenter studies should evaluate generalizability, sustainability, and clinical impact across diverse patient populations and ICU settings.

## Conclusion

In this single-center, prospective study of critically ill infants and children, the implementation of a self-tracking 15-task daily rounding checklist, designed to guide both the de-escalation of ICU support and the initiation of proactive measures, achieved acceptable compliance over a 300-day period. The checklist resulted in 198 documented plan modifications, capturing missed opportunities to optimize care, and thereby, supporting timely weaning of interventions and reducing iatrogenic risk.

These findings support the checklist's role in enhancing rounding processes, reducing iatrogenic risks, and embedding ICU liberation principles into daily pediatric critical care practice. Further research is needed to assess the broader clinical impact of this intervention, including its effects on outcomes such as mortality, hospital length of stay, and incidence of iatrogenic complications. This study offers a foundational step toward operationalizing structured, physician-led tools that are simple, scalable, and adaptable across institutions.

## Data Availability

The raw data supporting the conclusions of this article will be made available by the authors, without undue reservation.
